# Supplementary material for: The Lockdown Impact on the Relations between Portuguese Parents and Their 1- to 3-Year-Old Children during the COVID-19 Pandemic
Source: Children (Basel). 2022 Jul 28;9(8):1124. doi: 10.3390/children9081124 (PMC9406864; doi:10.3390/children9081124)
Supplement: Supplementary file 1 [file children-09-01124-s001.zip › children-1785626-supplementary.pdf]

Table S1. Univariable analysis for the outcome: Impact perceived by parents.

Variables	OR-estimate (95%CI)	*p*-value
Father[a]	1.25 (0.54 to 2.90)	0.609
Age of adult (years)	0.97 (0.93 to 1.02)	0.244
Marital status[b]		
Married / couple	1.35 (0.57 to 3.18)	0.491
Divorced / separated	0.72 (0.17 to 3.02)	0.649
Education: university[c]	0.53 (0.24 to 1.17)	0.114
Occupation[d]		
Self-employed	1.50 (0.74 to 3.04)	0.261
Unemployed	1.32 (0.41 to 4.28)	0.645
Other	0.69 (0.24 to 1.94)	0.477
Child age[e]		
2 year old child	0.60 (0.33 to 1.12)	0.109
3 year old child	0.44 (0.24 to 0.81)	0.008
Who took care of the child before the lockdown[f]		
Mother / father / both	2.49 (0.78 to 7.98)	0.126
Grandparents / other relatives	1.15 (0.52 to 2.55)	0.722
Time of contact with the child (hours) before the lockdown	1.10 (0.92 to 1.32)	0.309
Psychiatric history[g]		
Current psychiatric or psychologic follow-up	0.67 (0.42 to 1.07)	0.096
Past psychiatric or psychologic follow-up	0.61 (0.27 to 1.37)	0.234
Psychiatric medication	1.24 (0.63 to 2.42)	0.534
Number of children residing with the child in confinement	0.93 (0.75 to 1.15)	0.475
Number of adults residing with the child in confinement	1.36 (0.88 to 2.09)	0.162
Working place during the lockdown[h]		
Working from home	0.99 (0.50 to 1.98)	0.995
Lay off/already worked from home	0.88 (0.44 to 1.75)	0.712
Shared the care of child during confinement	1.52 (0.97 to 2.39)	0.067
Not stayed in the same address	0.86 (0.46 to 1.60)	0.631
Not maintained same household composition	0.74 (0.39 to 1.42)	0.365
Shared experiences with the child	2.84 (1.58 to 5.10)	<0.001
Evolution in the development of the child	10.49 (5.48 to 20.09)	<0.001
Regression in the child	0.26 (0.16 to 0.40)	<0.001
Interference of the child behavior in the daily tasks[i]		
Easier to handle tasks	0.62 (0.24 to 1.62)	0.621
Harder to handle tasks	0.18 (0.09 to 0.34)	0.176
PDHS: subscale challenging behavior	0.85 (0.81 to 0.89)	<0.001
PDHS: subscale parents' tasks	0.89 (0.86 to 0.92)	<0.001
PDHS: High Stress of Parents (FS)[j]	0.27 (0.18 to 0.42)	<0.001
PDHS: High Pressure on Parents (IS)[k]	0.30 (0.18 to 0.48)	<0.001

Note: PDHS: Parenting daily hassles scale; FS: frequency scale; IS: intensity scale. Reference categories: [a]mother; [b]single; [c]lower than university; [d]employee; [e]1 year old child; [f]kindergarten/babysitter; [g]no psychiatric history; [h]in the workplace; [i]there were no changes; [j]low stress of parents; [k]low pressure on parents.

Table S2. Univariable analysis for the outcome: Regressions in children development perceived by parents.

Variables	OR-estimate (95%CI)	*p*-value
Father[a]	0.93 (0.51 to 1.35)	0.451
Age of adult (years)	1.01 (0.98 to 1.03)	0.716
Marital status[b]		
Married / couple	0.73 (0.43 to 1.22)	0.228

Divorced / separated	0.84 (0.31 to 2.29)	0.726
Education: university[c]	0.94 (0.66 to 1.36)	0.757
Occupation[d]		
Self-employed	0.68 (0.46 to 1.02)	0.064
Unemployed	0.88 (0.46 to 1.68)	0.687
Other	1.45 (0.76 to 2.79)	0.261
Child age[e]		
2 year old child	2.30 (1.57 to 3.37)	<0.001
3 year old child	3.19 (2.18 to 4.67)	<0.001
Who took care of the child before the lockdown[f]		
Mother / father / both	0.61 (0.35 to 1.05)	0.074
Grandparents / other relatives	0.54 (0.31 to 0.94)	0.028
Time of contact with the child (hours) before the lockdown	0.97 (0.88 to 1.08)	0.618
Psychiatric history[g]		
Current psychiatric or psychologic follow-up	1.19 (0.69 to 2.04)	0.539
Past psychiatric or psychologic follow-up	1,40 (1.06 to 1.85)	0.018
Psychiatric medication	1.26 (0.89 to 1.79)	0.191
Number of children residing with the child in confinement	0.95 (0.82 to 1.09)	0.457
Number of adults residing with the child in confinement	0.92 (0.75 to 1.13)	0.426
Working place during the lockdown[h]		
Working from home	1.62 (1.03 to 2.55)	0.036
Lay off/already worked from home	1.68 (1.06 to 2.66)	0.026
Shared the care of child during confinement	0.85 (0.64 to 1.12)	0.239
Not stayed in the same address	1.18 (0.81 to 1.72)	0.387
Not maintained same household composition	1.09(0.72 to 1.65)	0.692
Positive impact in the relationship	0.26 (0.16 to 0.40)	<0.001
Shared new experiences with the child	0.52 (0.34 to 0.78)	0.002
Attitude and behavior of the child[i]		
More challenging harder to handle child	3.91 (2.80 to 5.45)	<0.001
More collaborative easier to handle child	1.15 (0.72 to 1.81)	0.565
PDHS: subscale challenging behavior	1.11 (1.09 to 1.14)	<0.001
PDHS: subscale parents' tasks	1.07 (1.05 to 1.10)	<0.001
PDHS: High Stress of Parents (FS)[j]	2.46 (1.90 to 3.20)	<0.001
PDHS: High Pressure on Parents (IS)[k]	3.13 (2.28 to 4.29)	<0.001

Note: PDHS: Parenting daily hassles scale; FS: frequency scale; IS: intensity scale. Reference categories: [a]mother; [b]single; [c]lower than university; [d]employee; [e]1 year old child; [f]kindergarten/babysitter; [g]no psychiatric history; [h]in the workplace; [i]there were no changes; [j]low stress of parents; [k]low pressure on parents.

Table S3. Univariable analysis for the outcome: Willingness to promote some change in family routines in the future.

Variables	OR-estimate (95%CI)	*p*-value
Father[a]	0.80 (0.58 to 1.12)	0.191
Age of adult (years)	0.98 (0.96 to 1.00)	0.061
Marital status[b]		
Married / couple	0.66 (0.42 to 1.03)	0.066
Divorced / separated	0.63 (0.29 to 1.40)	0.255
Education: university[c]	0.86 (0.65 to 1.13)	0.277
Occupation[d]		
Self-employed	1.05 (0.80 to 1.40)	0.744
Unemployed	1.14 (0.71 to 1.83)	0.588
Other	0.65 (0.39 to 1.11)	0.115
Child age[e]		
2 year old child	1.01 (0.80 to 1.27)	0.969
3 year old child	0.92 (0.73 to 1.18)	0.520
Who took care of the child before the lockdown[f]		
Mother / father / both	1.11 (0.78 to 1.58)	0.571

Grandparents / other relatives	1.15 (0.82 to 1.62)	0.408
Time of contact with the child (hours) before the lockdown	0.95 (0.88 to 1.03)	0.223
Psychiatric history[g]		
Current psychiatric or psychologic follow-up	1.52 (0.99 to 2.33)	0.054
Past psychiatric or psychologic follow-up	1.28 (1.03 to 1.59)	0.026
Psychiatric medication	1.25 (0.94 to 1.65)	0.122
Number of children residing with the child in confinement	0.97 (0.88 to 1.07)	0.540
Number of adults residing with the child in confinement	1.26 (1.07 to 1.47)	0.004
Working place during the lockdown[h]		
Working from home	1.38 (1.04 to 1.83)	0.027
Lay off/already worked from home	1.51 (1.13 to 2.02)	0.006
Shared the care of child during confinement	1.03 (0.84 to 1.27)	0.774
Not stayed in the same address	1.07 (0.80 to 1.43)	0.637
Not maintained same household composition	1.25 (0.90 to 1.72)	0.186
Shared new experiences with the child	3.07 (2.15 to 4.38)	<0.001
Attitude and behavior of the child[i]		
More challenging harder to handle child	1.58 (1.28 to 1.95)	<0.001
More collaborative easier to handle child	2.95 (2.23 to 3.91)	<0.001
PDHS: subscale challenging behavior	1.00 (0.99 to 1.02)	0.624
PDHS: subscale parents' tasks	1.01 (0.99 to 1.02)	0.261
PDHS: High Stress of Parents (FS)[j]	1.35 (1.09 to 1.67)	0.006
PDHS: High Pressure on Parents (IS)[k]	1.08 (0.81 to 1.44)	0.608
Father[a]	0.80 (0.58 to 1.12)	0.191

Note: PDHS: Parenting daily hassles scale; FS: frequency scale; IS: intensity scale. Reference categories: [a]mother; [b]single; [c]lower than university; [d]employee; [e]1 year old child; [f]kindergarten/babysitter; [g]no psychiatric history; [h]in the workplace; [i]there was no change in the childs' behavior; [j]low stress of parents; [k]low pressure on parents.
